# The Vietnam Initiative on Zoonotic Infections (VIZIONS): A Strategic Approach to Studying Emerging Zoonotic Infectious Diseases

**DOI:** 10.1007/s10393-015-1061-0

**Published:** 2015-09-24

**Authors:** Maia A. Rabaa, Ngo Tri Tue, Tran My Phuc, Juan Carrique-Mas, Karen Saylors, Matthew Cotten, Juliet E. Bryant, Ho Dang Trung Nghia, Nguyen Van Cuong, Hong Anh Pham, Alessandra Berto, Voong Vinh Phat, Tran Thi Ngoc Dung, Long Hoang Bao, Ngo Thi Hoa, Heiman Wertheim, Behzad Nadjm, Corina Monagin, H. Rogier van Doorn, Motiur Rahman, My Phan Vu Tra, James I. Campbell, Maciej F. Boni, Pham Thi Thanh Tam, Lia van der Hoek, Peter Simmonds, Andrew Rambaut, Tran Khanh Toan, Nguyen Van Vinh Chau, Tran Tinh Hien, Nathan Wolfe, Jeremy J. Farrar, Guy Thwaites, Paul Kellam, Mark E. J. Woolhouse, Stephen Baker

**Affiliations:** 1 Centre for Immunity, Infection & Evolution, The University of Edinburgh, Edinburgh, UK; 2 Oxford University Clinical Research Unit, Wellcome Trust Major Overseas Programme, Ho Chi Minh City, Vietnam; 3 Centre for Tropical Medicine, Nuffield Department of Clinical Medicine, Oxford University, Oxford, UK; 4 Global Viral, San Francisco, USA; 5 The Wellcome Trust Sanger Institute, Cambridge, UK; 6 Oxford University Clinical Research Unit, Wellcome Trust Major Overseas Programme, Hanoi, Vietnam; 7 The Hospital for Tropical Diseases, 764 Vo Van Kiet, Quan 5, Ho Chi Minh City, Vietnam; 8 Laboratory of Experimental Virology, Center for Infection and Immunity Amsterdam (CINIMA), Academic Medical Center of the University of Amsterdam, Amsterdam, The Netherlands; 9 Hanoi Medical University, Hanoi, Vietnam; 10 The London School of Hygiene and Tropical Medicine, London, UK

**Keywords:** zoonotic infection, Vietnam, high-risk cohort, disease surveillance, diseases of unknown origin, diagnostics, ultra-deep sequencing, genomics, social science

## Abstract

The effect of newly emerging or re-emerging infectious diseases of zoonotic origin in human populations can be potentially catastrophic, and large-scale investigations of such diseases are highly challenging. The monitoring of emergence events is subject to ascertainment bias, whether at the level of species discovery, emerging disease events, or disease outbreaks in human populations. Disease surveillance is generally performed *post hoc*, driven by a response to recent events and by the availability of detection and identification technologies. Additionally, the inventory of pathogens that exist in mammalian and other reservoirs is incomplete, and identifying those with the potential to cause disease in humans is rarely possible in advance. A major step in understanding the burden and diversity of zoonotic infections, the local behavioral and demographic risks of infection, and the risk of emergence of these pathogens in human populations is to establish surveillance networks in populations that maintain regular contact with diverse animal populations, and to simultaneously characterize pathogen diversity in human and animal populations. Vietnam has been an epicenter of disease emergence over the last decade, and practices at the human/animal interface may facilitate the likelihood of spillover of zoonotic pathogens into humans. To tackle the scientific issues surrounding the origins and emergence of zoonotic infections in Vietnam, we have established The Vietnam Initiative on Zoonotic Infections (VIZIONS). This countrywide project, in which several international institutions collaborate with Vietnamese organizations, is combining clinical data, epidemiology, high-throughput sequencing, and social sciences to address relevant one-health questions. Here, we describe the primary aims of the project, the infrastructure established to address our scientific questions, and the current status of the project. Our principal objective is to develop an integrated approach to the surveillance of pathogens circulating in both human and animal populations and assess how frequently they are exchanged. This infrastructure will facilitate systematic investigations of pathogen ecology and evolution, enhance understanding of viral cross-species transmission events, and identify relevant risk factors and drivers of zoonotic disease emergence.

## Background

The burden of infectious diseases in human populations in low- and middle-income countries remains high (Sepúlveda and Murray 2014); in the majority of cases, disease etiologies are never determined (Kotloff et al. 2012; Mulholland 2003; Susilawati and McBride 2014), often due to inadequate laboratory diagnostic capacity. The causative agents of diseases of unknown origin (DUOs) fall broadly into three groups: (1) known pathogens that were not tested for or misdiagnosed; (2) previously unrecognized but common pathogens (e.g., parechoviruses and human metapneumovirus); and (3) newly emerging or re-emerging pathogens (e.g., filoviruses, henipaviruses, and coronaviruses). All three categories are relevant to policymakers when trying to allocate resources for prevention. While the third category accounts for the smallest number of infections, the impact of emerging pathogens, particularly those with potential for human-to-human transmission, may range from disruptive to catastrophic. Developing an infrastructure to tackle these issues is challenging and costly, yet interventions that are implemented without supporting data on DUO frequency and population risk may prove ineffective.

Newly emerging pathogens in low- to middle-income countries are likely to originate from an animal source. Approximately 60% of all human pathogen species are known to be zoonotic (Woolhouse and Gowtage-Sequeria 2005) and pathogens that infect multiple species are three times as likely to emerge into human populations than host-restricted pathogens (Taylor et al. 2001). Some pathogens undergo enzootic transmission in reservoir animal populations with occasional spillover into a human host and little to no onward transmission (Jonsson et al. 2010; Wertheim et al. 2009). Other pathogens display human-to-human transmission after spillover and may result in large epidemics (Drosten et al. 2013; Gire et al. 2014; Janies et al. 2008). Some of these emerging pathogens may eventually adapt to circulate exclusively among humans, leading to epidemic or endemic transmission cycles (Holmes and Twiddy 2003; Taubenberger 2006). The frequency with which such zoonotic transmission events occur and the likelihood that a pathogen will adapt to exclusively human transmission are largely determined by behavioral and immunological factors in the host, along with ecological and evolutionary factors of the pathogen (Karesh et al. 2012).

It has been proposed that the focus of zoonosis research should move toward the early detection of zoonotic pathogens, in particular those that exhibit potential for emergence in humans (Morse et al. 2012). This is particularly poignant given the current epidemic of Ebola virus in Western Africa, where sustained animal/human surveillance prior to the start of the outbreak may have had an impact on the scale of the epidemic.

Large-scale investigations of emerging zoonotic infections are challenging. Monitoring is subject to ascertainment biases, whether at the level of species discovery, emerging disease events, or disease outbreaks in human populations. Disease surveillance is often performed *post hoc*, driven by a response to recent events and by the availability and sustainability of detection technologies. Additionally, the inventory of pathogens that exist in mammalian and other reservoirs is astonishingly incomplete (Anthony et al. 2013) and identifying those with the potential to cause disease in humans is rarely possible in advance. Finally, the nature of the species barrier and the factors that enable pathogens to cross these barriers and establish transmission among humans are largely unknown.

The factors mentioned above limit our ability to study zoonotic pathogens in detail; therefore, we identified the need for active monitoring of human and animal reservoirs of infection to characterize community exposures and infections in a developing country with a prolonged history of zoonotic transmission events and outbreaks. The two major requirements for understanding the burden and diversity of zoonotic infections and the behavioral/demographic risks of infection are the establishment of surveillance networks in populations that maintain regular contact with diverse animal populations and the simultaneous characterization of pathogen diversity in human and animal populations. Here, we describe a project that is currently underway in communities across Vietnam in which we are collecting clinical samples and associated clinical, epidemiological, and demographic data, which will be combined with high-throughput viral genome sequences and qualitative social sciences data to address key one-health questions with the aim of better understanding the origins, risks, and emergence of zoonotic infections.

## The Vietnam Initiative on Zoonotic Infections (VIZIONS)

Southeast Asia is a global hotspot for emerging infectious diseases (Morse et al. 2012), and Vietnam has been an epicenter of emerging disease activity over the last decade (Dinh et al. 2006; Reynolds et al. 2006; Vinh et al. 2009; Vu Tra My et al. 2014; Wertheim et al. 2009). Vietnam has a large population (89,700,000 in 2013 (*Statistical yearbook of Vietnam*, 2013)), some of the highest livestock densities in Southeast Asia (Gerber et al. 2005), and a substantial burden of DUOs (Ho Dang Trung et al. 2012; Thompson et al. 2015). Furthermore, approximately 50% of the Vietnamese population reside in rural areas and participate in small-scale animal production (*Statistical yearbook of Vietnam*, 2013).

We hypothesize that Vietnam’s demography, varied animal production systems, and food consumption habits facilitate the spillover of zoonotic pathogens into humans. Specifically, we predict that exotic food production systems with mixed species and limited biosecurity, abattoirs and wet markets operating with minimal basic hygiene, poor cold chains for meat distribution, limited meat inspections in the market sector, and consumption of raw/undercooked blood, meat, organ tissues, and wild animal products promote the risk of zoonotic pathogen transmission.

VIZIONS, initiated in March 2012, is now an established platform for one-health research in Vietnam. With VIZIONS, we are aiming to integrate traditional clinical, epidemiological, and medical anthropological methods with new approaches for pathogen detection and discovery, including novel sequencing approaches combined with phylogenetic analysis to characterize pathogen populations. The principal aims of VIZIONS are presented in Box 1.

**Box 1:** The principal aims of the VIZIONS project

1. To establish a model international collaborative consortium with an integrated approach to human and animal health research
2. To estimate the burden of disease (focusing on viral and zoonotic diseases), and investigate the disease epidemiology in patients hospitalized with specified clinical syndromes and infections in a cohort of high-risk individuals occupationally exposed to animals; with targeted sampling from domestic animals and wildlife in association with these individuals
3. To elucidate the etiology of infectious diseases of unknown origin in the human population, and provide a repository of putative pathogens for further study
4. To characterize genetic diversity within virus populations on either side of the species barrier in order to understand cross-species transmission and disease emergence
5. To identify socio-demographic, environmental, and behavioral drivers for disease emergence
6. To create a platform and resource for further research on zoonotic disease agents

**Figure 1:**
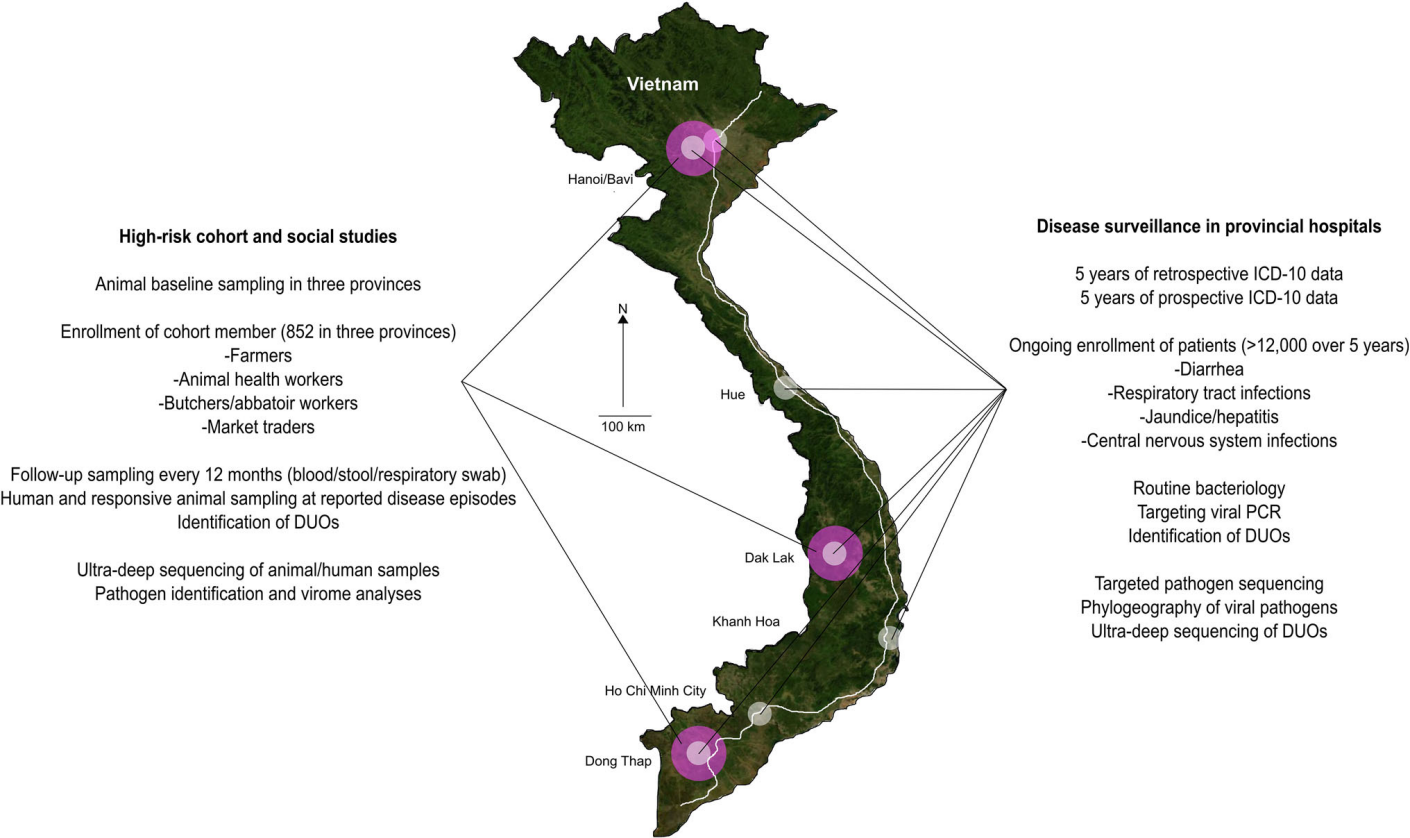
The structure of the VIZIONS project across Vietnam. Map of Vietnam showing the main components of VIZIONS, these are (1) hospital-based surveillance program studying respiratory tract infections, central nervous system infections (CNS), enteric infections, and jaundice for detailed clinical and epidemiological investigation; in Dong Thap, Ho Chi Minh City, Dak Lak, Khanh Hoa, Hue, Hanoi, and Ba Vi (*small white circles*) and (2) a longitudinal cohort study of occupational risk of zoonotic infections, plus records of risk behaviors, with linked sampling of putative animal reservoirs that will generate >50,000 specimens for molecular investigation being performed in Dong Thap, Dak Lak, Khanh Hoa, and Ba Vi (*large pink circles*).

To deliver the aims of VIZIONS, we established two fundamental components: a hospital disease surveillance program to characterize endemic infections, novel infections and DUOs, and a high-risk sentinel zoonosis cohort to assess disease incidence and pathogen transfer (Figure 1).

## Hospital Disease Surveillance

The hospital surveillance component of VIZIONS is underway in seven locations. These locations are shown in Figure 1, and collaborating Vietnamese institutions are outlined in Box 2. Over a 3-year period of enrollment at each site, we aim to enroll up to 3000 hospitalized cases of each of four key clinical syndromes (central nervous system (CNS) infections, enteric infections, jaundice, and respiratory tract infections) that may be caused by a zoonotic pathogen. The aim to enroll 3000 cases under each clinical syndrome was based both on operational capacity and local epidemiology, specifically the ability to calculate population attributable fractions (PAFs) for specific pathogens (thus providing supporting evidence of disease etiology). These samples sizes were considered to be sufficient to estimate PAFs to informative levels of precision: for example, a pathogen found in 20% of 3000 cases and 40% of 1000 severe cases will give PAF = 33% with approximate 95% confidence intervals of 30–37%. From pilot data, we anticipated identifying a pathogen associated with the defined clinical syndromes in 50% of CNS cases, 60% of respiratory cases, 60% of enteric cases, and 30% of hepatitis cases; roughly 50–80% of these pathogens were predicted to be viruses. These enrollment targets should provide samples and metadata from known infections and provide >80% power to detect a pathogen that is present in just 1/1800 patients with a specified syndrome.

**Box 2:** Key VIZIONS institutions, organizations, and collaborations within Vietnam

Hospitals
The Hospital for Tropical Diseases (HTD), Ho Chi Minh City
Dong Thap General Hospital, Cao Lanh City, Dong Thap Province
Dak Lak General Hospital, Buon Ma Thuot City, Dak Lak Province
Khanh Hoa General Hospital, Nha Trang City, Khanh Hoa Province
Hue Central Hospital, Hue City, Thua Thien Hue Province
National Hospital for Tropical Diseases (NHTD), Ha Noi
Ba Vi District Hospital, Ha Noi
Academic institutions
Oxford University Clinical Research Unit (OUCRU), Ho Chi Minh City
Oxford University Clinical Research Unit (OUCRU), Ha Noi
Hanoi Medical University (HMU)
Regional Animal Health Office (RAHO)
RAHO 5, Buon Ma Thuot City, Dak Lak Province
Sub-departments of Animal Health (sDAH)
Dak Lak sDAH, Buon Ma Thuot City, Dak Lak Province
Dong Thap sDAH, Cao Lanh City, Dong Thap Province
Preventive Medicine Centres (PMC)
Dak Lak PMC, Buon Ma Thuot City, Dak Lak Province
Dong Thap PMC, Cao Lanh City, Dong Thap Province
Ba Vi District PMC, Ha Noi
Ba Vi District Veterinary Station

**Figure 2:**
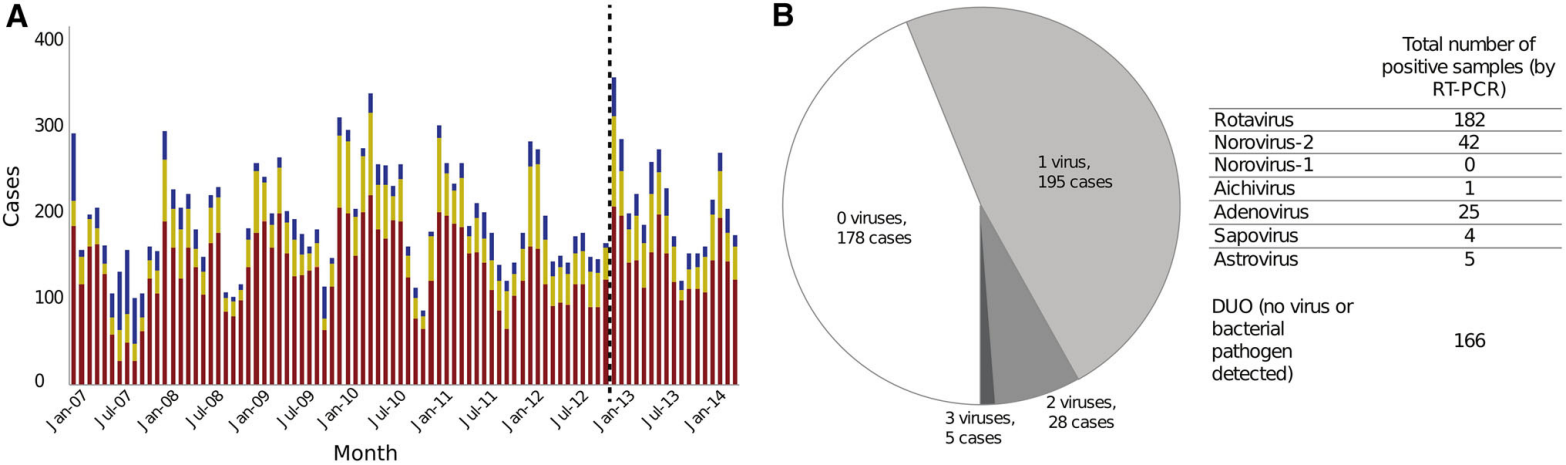
Diarrheal disease at Dong Thap Provincial Hospital, 2007–2014. **a** Hospital case data showing the number of hospital visits (outpatient and inpatient) for diarrheal disease under the three most common ICD-10 codes used in this hospital (representing 99.4% of all diarrheal disease records) from January 2007 to April 2014. *Red* indicates cases classified under ICD-10 code *A04* (other bacterial intestinal infections), *yellow* indicates cases classified as *A08* (viral and other specified intestinal infections), and *blue* indicates cases classified as *A09* (diarrhea and gastroenteritis of presumed infectious origin). The *dashed line* shows the initiation of VIZIONS hospital surveillance in Dong Thap Provincial Hospital, November 2012. **b** Number of viruses detected in diarrheal disease samples collected under VIZIONS protocols from November 2012 through April 2014. Screening included RT-PCR for the listed panel of viral pathogens, as well as standard microbiological culture (data not shown). A diarrheal disease DUO is designated as a sample in which no pathogen was detected by the aforementioned screening methods.

Upon enrollment and informed consent, data including diagnostic investigations (clinical and laboratory), age, sex, occupation, animal exposure, residential address, household size, income, and wealth indicators are recorded via a standardized case report form. The residence of each case is geo-located and will ultimately be associated with an existing suite of spatial datasets. Additionally, we are collecting routine data for all hospital admissions (retrospectively and prospectively) via the International Classification of Diseases 10 (ICD-10) and are modeling hospital catchment populations by comparing the spatial distribution of VIZIONS-enrolled patients to the entire population entering the healthcare facilities. These data are being used further to predict atypical patterns of hospital admissions to detect outbreaks (Figure 2).

Patient samples (listed according to disease syndrome: CNS, cerebrospinal fluid (CSF) and blood/plasma; enteric, stool; jaundice, blood/plasma; respiratory, sputum/nasopharyngeal swab) are collected and subjected to a bacteriological and viral diagnostic algorithm for each clinical syndrome. This diagnostic process is conducted within Vietnam (both at study sites and at Oxford University Clinical Research Unit (OUCRU) laboratories) and was designed to identify the major known causes of the four specific syndromes of interest. Cases are subsequently categorized as being associated with a known causative agent or as a DUO. At the time of writing (May 2015), we have recruited >6500 patients across the four syndromes.

## The High-Risk Sentinel Cohort

**Figure 3:**
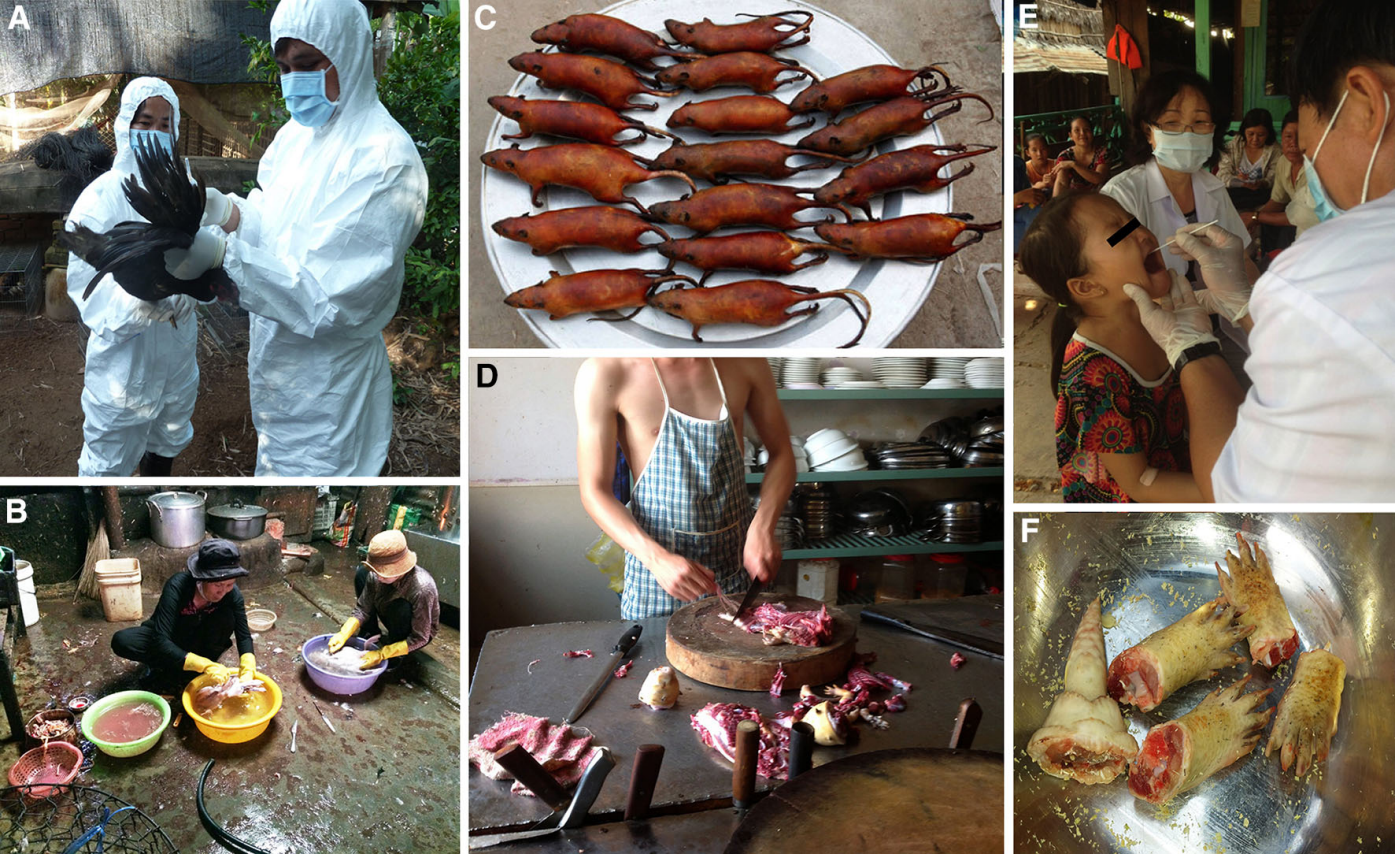
The VIZIONS high-risk cohort in Dong Thap, Dak Lak, and Ba Vi. The second major operational component of VIZIONS is sampling a high-risk cohort of people that may be likely to be hosts of zoonotic pathogen transfer as a consequence of occupational exposure to animals. This component of the study is being performed in three locations (Dong Thap, Dak Lak, and Ba Vi). These photographs outline some of the main activities and locations related to this cohort. **a** Responsive animal sampling with the sub-department of animal health in Dong Thap province, **b** Market workers cleaning poultry in Dak Lak province, **c** Rats prepared for human consumption in Dong Thap province, **d** A restaurant worker butchering bamboo rat meat in Dak Lak province, **e** Sampling a child enrolled in the cohort study on a farm in Dong Thap province, and **f** Butchered porcupine feet prepared for consumption in Dak Lak province.

Working with local academic and governmental partners in three provinces (Figure 1, Box 2), we have additionally established a high-risk, sentinel cohort. We have recruited 880 individuals that we consider to be at high-risk for zoonotic pathogen transfer as a consequence of occupational exposure to animals (Figure 3). Cohort members were selected from (1) farming households, especially those with mixed livestock and wildlife species, (2) wildlife restaurants, (3) abattoirs, (4) wet markets, and (5) other high-risk occupational groups such as animal health workers/veterinarians and wildlife trappers/traders. Potential cohort members were identified and screened for suitability and, after providing informed consent, were asked to provide a blood sample, nose and throat swab, and a rectal swab, followed by the administration of a questionnaire to document socioeconomic factors, health-seeking behavior, occupational hazards, animal exposure, and food consumption habits. Participants are then interviewed and sampled annually to document risk factors for zoonotic infections, such as animal exposure, food consumption habits, and disease episodes within their household or among their animals. Throughout the three-year study, participants who develop illness are encouraged to visit the local study hospital or are visited at home by a member of the local Preventative Medicine Centre (PMC). The medical workers determine whether the illness is likely to represent an infectious disease episode and, if so, collect clinical samples from the cohort member and administer a disease episode questionnaire related to animal exposures. Additionally, the local sDAH are informed and a team of animal health workers visits the site within 48 hours of the reported disease episode for responsive sampling of representative animal species present at the study site (Figure 3).

This longitudinal cohort component of VIZIONS is designed to enable detection and monitoring of cross-species virus ‘chatter’ between animal and human populations. Further, these repositories of linked human and animal specimens will permit us to determine the incidence and seroprevalence of selected zoonoses within the cohort, define viral diversity in humans with different animal exposures and in representative non-human species, and characterize human–animal contact behavior in a variety of settings. Such behavior considered in the context of this project includes the reported slaughter, butchering, and rendering of livestock and wildlife, handling or consumption of diseased animals, and consumption of raw animal products. These data and specimen repositories are expected to provide a resource for the identification of novel or unexpected agents of human disease and their potential zoonotic origins, and to deliver information that can be used to examine the barriers to and drivers of cross-species transmission.

## Genomics and Phylogenetics

Phylogenetic studies have been utilized to investigate endemic and emerging pathogen populations at various scales and offer significant insight into the evolutionary and epidemiological characteristics of pathogens involved in disease emergence (Grenfell et al. 2004; Pybus and Rambaut 2009). Within the context of VIZIONS, we are performing full and partial genome sequencing to characterize both ubiquitous and novel viral populations present in Vietnam. Using these sequences, we aim to identify principal zoonotic viral populations and investigate their dynamics in human and animal populations. Initial pathogens prioritized for investigation include rotavirus, hepatitis E virus, influenza A virus, and enteroviruses. For these and other viral populations that are found to be endemic in Vietnam or show extensive transmission, we will use phylogenetic methods to characterize their spatial and temporal spread through animal and/or human populations; these investigations will provide information on large- and small-scale networks of disease transmission within the country, with which we hope to gain a better understanding of the patterns and determinants of pathogen dispersal.

In the case of DUOs, where no pathogen is detected by standard screening methods, metaviral sequencing methods (Cotten et al. 2014) will be used to identify viral nucleic acids within the sample. To provide further context on the extent of viral exchange between human and animal populations and characterize viral diversity across species of interest, we will also use metagenomic methods to characterize the viromes of healthy human and animal specimens. Viruses that show zoonotic potential in these populations will be further investigated through population-level serological studies to determine the extent and risks of these zoonotic infections across Vietnam.

## Social Sciences

Anthropogenic factors of expanding peri-urban regions, increased natural resource use, changing scales of agriculture, and human encroachment into wildlife habitat have led to the development of intensive agricultural practices and exotic species farming in Vietnam. These factors increase the frequency, duration, and intensity of wildlife, livestock, domestic animal, and human interaction (Rhyan and Spraker 2010). Vietnam now has unprecedented demands for meat from livestock, and many wildlife species are now commonly farmed (e.g., bamboo rat, wild boar, civet, porcupine) (Brooks et al. 2010; Drury 2009). The maintenance of large animal populations (domestic and wildlife species) generally involves supplemental feeding and the introduction of animals from other populations—all factors that facilitate pathogen transmission (Wildlife Conservation Society 2008). Additionally, the presence of mixed wildlife and livestock populations on farms increases the risk of introduction of novel pathogens to potentially immunologically naive populations. Although human exposure to livestock is known to be an important risk factor for cross-species transmission of zoonotic pathogens in this region (Dinh et al. 2006; Ho et al. 2011; Mackenzie 2005), the risk posed by wildlife remains unknown; this is due partly to an incomplete understanding of wildlife farming and consumption practices.

We aim to investigate the socio-cultural context of wildlife consumption and farming within a subset of cohort participants with exposure to wildlife. In parallel to the annual participant interviews, we are conducting qualitative research including participant observation and in-depth interviews to assess contextual and behavioral risk factors in a subset of individuals with specific exposures to wildlife species, including wildlife farmers, trappers, traders, and restaurant workers.

## Current Status, Lessons Learned, and Sustainability

At the time of writing, the hospital component of the VIZIONS project is running to schedule with respect to data collection, patient recruitment, and pathogen screening. This has been a substantial resource for collaborating hospitals, where laboratory diagnoses are seldom performed and infections are typically identified through standard clinical observations. Therefore, we are developing a thorough understanding of the disease burden of many key pathogens across Vietnam but still have an alarming rate of DUOs, ranging from 42.6% (764 of 1792 samples screened) in diarrhea cases to 74.7% (464 of 621 samples screened) in CNS cases (2451/4624 cases overall across the four syndromes have no identified pathogen). While these results are not reported back to hospitals in real time, allowing an immediate improvement of clinical assessment, they are providing vital seasonal information regarding annual fluctuations in hospital attendance and disease etiology. A key lesson from the hospital component of the study has been understanding and accounting for the gaps in diagnostic capabilities for patients who present with a disease of a presumed infectious origin in the hospital setting. A lack of diagnostic technology and funding to perform adequate diagnostics is a major issue in the healthcare systems of many developing countries and leaves treating clinicians to make diagnoses based on medical intuition and experience alone. This impacts the quality and consistency of data coming in from hospital sites, requiring additional considerations and generalizations to be made in our analyses, particularly those dependent on epidemiological data collected directly from the hospitals. Furthermore, even though we are performing a considerable range of microbiological and molecular screening panels for major pathogens, the rate of DUOs is still high. By working in further detail on these DUO specimens, we hope to uncover previously undiagnosed pathogens, thus reducing the proportion of patients hospitalized with DUOs. A better understanding of regional pathogens causing infections should, eventually, lead to better diagnostic approaches, which ideally should be performed in a multiplex system in a timeframe that is relevant to patient care and cost-effective for use in developing country settings.

With respect to the cohort component of the VIZIONs project, we are currently following 880 individuals in three provinces; all of these individuals have now been in the cohort for at least 1 year. To date, we have recorded 532 disease episodes in these individuals, mainly manifesting as fever, diarrhea, or respiratory infections. The collections of samples (from both routine and disease sampling) from this component of the study are, at this scale, unique and many are currently in a metaviral nucleic acid sequencing pipeline. We are currently extracting and screening over 3000 fecal specimens from humans and animals that are included in the cohort study using these methods. Further, we are screening for a range of endemic zoonotic pathogens including influenza, hepatitis E, rotavirus, and calciviruses. The cohort component of VIZIONs will be complete in mid-2017.

The formation and maintenance of VIZIONs has been a substantial nationwide effort, but we think that the nature of the now established network will be productive and will yield a major resource for understanding zoonotic infections in Vietnam. Probably the greatest lesson learned has been in getting the various governmental and non-governmental organizations to work in unison. In Vietnam, like in many other countries, governmental and provincial departments supporting animal and human health largely work in administrative silos, with little overlap in their political direction or interests. The relationships that have been created between the independent government departments and academic research institutions will be key for the long-term sustainability of this network. However, as a consequence of zoonotic disease outbreaks occurring relatively infrequently, continual governmental funding for such a resource is often overlooked. Nevertheless, given our engagement with the various governmental departments and members of the Vietnamese public, we think that maintaining this infrastructure is both practical and financially viable. Through training schemes for laboratory workers, medical staff, community healthcare workers, and veterinarians, we are providing an ongoing capacity-building role and have provided support for several potential outbreaks and atypical clinical presentations in both the human and animal communities. Conversely, we believe that assessing the amount of zoonotic transfer and identifying pathogens likely to have an impact on human health will be the evidence required to persuade continued investment.

## Summary

Here we have described an established multidimensional platform in Vietnam aimed to tackle scientific issues surrounding the origins and emergence of zoonotic infections. This countrywide project, in which several international institutions collaborate alongside Vietnamese organizations, is combining clinical data, epidemiology, high-throughput sequencing, and social sciences to address key one-health questions that cannot be addressed independently. Our overarching objective is to develop an integrated and sustainable approach to the surveillance of pathogens circulating in both human and animal populations. This infrastructure will facilitate systematic investigations of pathogen ecology and evolution, enhance the understanding of viral cross-species transmission events, and allow us to identify the relevant risk factors and drivers of zoonotic disease emergence. The capacity of VIZIONS to detect and characterize unusual disease events in nearly real time will facilitate a new approach to protecting public health in Vietnam.
